# Integrated analysis of endometrial stromal cell long noncoding RNA and mRNA expression profiles associated with TGF-β1-induced fibrosis: lncRNA and mRNA expression profiles associated with TGF-β1-induced fibrosis in ESCs

**DOI:** 10.3724/abbs.2024052

**Published:** 2024-04-18

**Authors:** Jianhong Wu, Linyuan Fan, Lin Li, Yudi Zhang, Yucui Tian, Ziwen Jiang, Zhaohui Liu, Dan Lu, Yinmei Dai

**Affiliations:** 1 Department of Gynecology Beijing Obstetrics and Gynecology Hospital Capital Medical University Beijing Maternal and Child Health Care Hospital Beijing 100026 China; 2 Central Laboratory Beijing Obstetrics and Gynecology Hospital Capital Medical University Beijing Maternal and Child Health Care Hospital Beijing 100026 China

Intrauterine adhesions (IUAs) are a form of uterine fibrotic scar tissue that develops in response to traumatic injury to the endometrial basal layer. Patients with IUAs can suffer from menstrual abnormalities and various obstetric complications [1]. The formation of IUAs is largely driven by endometrial fibrosis, in which excessive amounts of extracellular matrix (ECM) proteins such as collagen type I alpha 1 chain protein (COL1A1) and α-smooth muscle actin (α-SMA) are deposited in the endometrium [2]. During endometrial regeneration, stromal cells undergo a stromal-to-epithelial transition that gives rise to epithelial tissue [3]. Under fibrosis-related conditions, however, endometrial stromal cells (ESCs) transdifferentiate into myofibroblasts via epithelial-mesenchymal transition (EMT). Transforming growth factor-β1 (TGF-β1) is a cytokine that induces fibrotic activity. Previous studies on IUA tissue samples from patients revealed that TGF-β1 is upregulated at the mRNA and protein levels compared with those in healthy controls, suggesting that it may be an important mediator of IUA progression [ 4, 5]. Efforts to target TGF-β1 may thus provide an avenue through which endometrial fibrosis can be prevented or treated. However, TGF-β1 also plays a variety of other important regulatory roles through the regulation of cell growth, migration, apoptotic death, and differentiation and is not the only mediator of fibrotic activity [6]. The suppression of TGF-β1 thus has the potential to cause substantial off-target toxicity, underscoring the need for the development of alternative approaches for the inhibition of endometrial fibrosis. Long noncoding RNAs (lncRNAs) serve as key regulators of diverse cellular processes, such as proliferation, apoptosis, and differentiation, through their ability to regulate gene expression. Recent evidence has highlighted the important roles of specific lncRNAs in shaping IUA development [7]. However, relatively few studies to date have comprehensively assessed the lncRNA expression profiles in IUA patients, and the therapeutic relevance of these lncRNAs has yet to be clarified.

In a prior study, we successfully utilized TGF-β1 to promote ESC myofibroblast transdifferentiation *in vitro*, thereby establishing a cellular model of IUA [8]. In the present study, we explored the lncRNA and mRNA expression profiles and the interactions associated with EMT induction in these TGF-β1-stimulated ESCs, and verified the key lncRNA-mRNA interaction pairs through this approach via qPCR and functional assays.

RNA-seq analyses of TGF-β1-treated ESCs revealed 327 significant DE-mRNAs and 366 significant DE-lncRNAs compared to control unstimulated ESCs. The top 20 upregulated and downregulated DE-mRNAs and DE-lncRNAs are shown in Supplementary Tables S1 and S2. Differences in the expressions of these transcripts were compared between groups using scatterplots ( Supplementary Figure S1A) and volcano plots ( Supplementary Figure S1B). In addition, heatmap clustering analyses were employed to systematically assess patterns of differential lncRNA and mRNA expressions between these two groups ( Supplementary Figure S1C). These analyses revealed that both lncRNA and mRNA expression patterns varied markedly when comparing ESCs that were and were not stimulated with TGF-β1.

To evaluate the possible functional roles that the DE-mRNAs may play in EMT induction in ESCs, functional enrichment analyses were conducted. The most enriched GO terms and KEGG pathways are listed in Supplementary Figure S2. In particular, the most enriched KEGG pathways included PI3K/Akt signaling, transcriptional misregulation in cancer, and the JAK/STAT signaling pathway, as well as pathways associated with cellular senescence, phagosomes, inflammatory mediator regulation of TRP channels, Toll-like receptor signaling, and Hippo signaling. To clarify which DE-mRNAs are likely to serve as core mediators of EMT induction, a PPI network composed of 149 nodes and 189 edges was constructed ( Supplementary Figure S3A). A subnetwork including the 20 DE-mRNAs with the highest connectivity in the PPI network was also identified ( Supplementary Figure S3B). This subnetwork included 16 upregulated and 3 downregulated DE-mRNAs after the exclusion of HSD17B7, as it is not directly connected to any of the other DE-mRNAs. The top 20 most connected genes, in order of degree of connectivity, are *IL6*, *CXCL8*, *IGF1*, *SOX9*, *SMC3*, *AREG*, *IGFBP3*, *IGF2*, *COL1A2*, *IHH*, *FDFT1*, *HSD17B7*, *TLR2*, *IL11*, *CNTF*, *KIF20B*, *IL21R*, *C4A*, *TPR*, and *ESF1*. These genes are regarded as hub genes that are likely to serve as the most important regulators of EMT induction in the context of IUA development.

To explore the interactions between DE-lncRNAs and DE-mRNAs, a lncRNA-mRNA network based on three potential modes of interaction between these transcripts was constructed. In total, 79 pairs of lncRNAs and mRNAs potentially linked through cis-regulatory mechanisms were identified, as well as 6643 pairs potentially linked through trans-regulatory mechanisms, and 11,246 pairs were identified based on the calculated binding free energy values between individual lncRNA and mRNA targets. Of these, 601 lncRNA-mRNA pairs were found to overlap across all three datasets ( Supplementary Figure S4A) and were used to establish a putative regulatory network ( Supplementary Figure S5). This network revealed that individual lncRNAs are potentially associated with multiple mRNA targets, as specific mRNAs are tentatively linked to multiple lncRNAs. Complex lncRNA-mRNA interactions are thus likely to play a central role in shaping EMT induction in the context of endometrial fibrosis. A subnetwork containing the top 8 DE-lncRNAs associated with the greatest number of target mRNAs was also extracted ( Supplementary Figure S4B). These top 8 lncRNAs include ZNNT1, BGIG9606_58128, BGIG9606_46964, LOC100129503, LOC102724814, YTHDF3-AS1, BGIG9606_36158, and BLOC1S5-TXNDC5.

The above results were subsequently used for the construction of a lncRNA-mRNA-pathway network ( Figure 1A). This network includes three TGF-β1 signaling-related pathways, the PI3K/Akt, JAK/STAT, and Hippo signaling pathways. The network incorporates 21 lncRNA-mRNA pairs, including 17 lncRNAs and 10 mRNAs. Of the included lncRNAs, ZNNT1, BGIG9606_58128, and LOC100129503 are among the eight lncRNAs that were found to be associated with the greatest numbers of mRNA targets. Moreover, of the included mRNAs, AREG, IGF2, COL1A2, IL11, and IL21R are among the top 20 mRNAs found to have the highest connectivity values. The lncRNA ZNNT1 is associated with four target mRNAs (EREG, LAMA1, WTIP, and IL11) as well as the PI3K/Akt, JAK/STAT, and Hippo signaling pathways. The expression patterns of these lncRNAs and mRNAs were then verified using qPCR in 10 IUA tissue samples and 10 normal endometrial tissue samples, confirming that 7 lncRNAs and their target mRNAs were differentially expressed following TGF-β1 treatment, consistent with the RNA-seq data ( Figure 1B,C) and confirming their reliability. The primers used are listed in Supplementary Table S3. This study received approval from the Ethical Committee of Beijing Obstetrics and Gynecology Hospital (approval No. 2022-KY-056-01), and all patients provided written informed consent.

**Figure 1:**
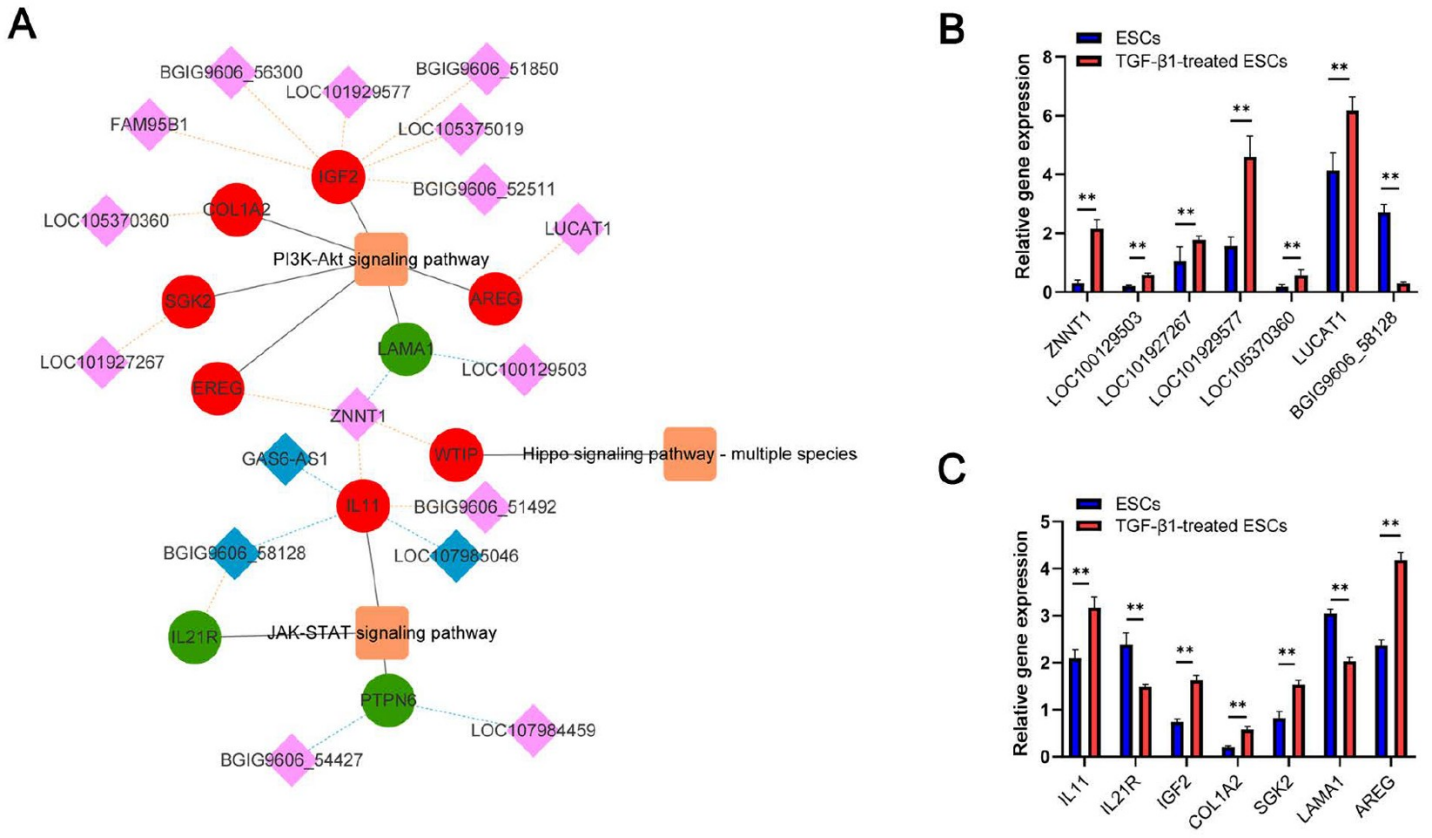
Figure 1 LncRNA-mRNA-pathway network and qPCR verification lncRNA-mRNA-pathway co-expression network. Red and green circular nodes represent upregulated and downregulated mRNAs, respectively, in TGF-β1-treated ESCs. Pink and blue diamond nodes represent upregulated and downregulated lncRNAs in TGF-β1-treated ESCs. Positive and negative correlations are indicated by yellow and light blue dotted lines, respectively. The gray edges indicate the participation of the indicated mRNAs in the indicated pathway. (B) qPCR-based verification of 7 lncRNAs in the lncRNA-mRNA-pathway network in TGF-β1-treated ESCs. Data are presented as the mean±SEM. **P<0.01 vs ESCs; unpaired Student’s t tests. (C) qPCR verification of associated mRNAs targeted by the 7 lncRNAs in TGF-β1-treated ESCs. Data are shown as the mean±SEM. **P<0.01 vs ESCs; unpaired Student’s t tests.

Given that ZNNT1 is associated with the greatest number of target mRNAs and is included in the core lncRNA-mRNA-pathway network established above, ZNNT1 was selected as a target for additional verification. Among the 4 ZNNT1-targeted mRNAs identified in the literature, IL11 mRNA is the most strongly related to fibrotic disease [ 9, 10], so the ZNNT1-IL11 interaction pair was selected for further verification. The expression of ZNNT1, as well as the levels of IL-11 and the fibrotic marker proteins COLA1 and α-SMA, were significantly elevated in IUA patient samples compared to those in control endometrial tissue samples ( Figure 2A,B). The qPCR results showed that the mRNA expression of IL11 changed commensurately with that of ZNNT1 ( Figure 2C). However, ZNNT1 mRNA expression did not change significantly in the si-IL11 groups ( Figure 2D), confirming that ZNNT1 positively and unidirectionally regulates IL11. To further investigate the regulatory role of ZNNT1, a dual-luciferase reporter assay was performed, which showed that *ZNNT1* knockdown suppressed IL11 luciferase activity ( Figure 2E), consistent with ZNNT1 acting as a posttranscriptional regulator of IL11. The protein levels of IL11, COL1A1, and α-SMA were also decreased significantly in TGF-β1-treated ESCs after *ZNNT1* silencing ( Figure 2F). These results confirmed that *ZNNT1* silencing can protect against endometrial fibrosis at least in part through the regulation of IL11 expression.

**Figure 2:**
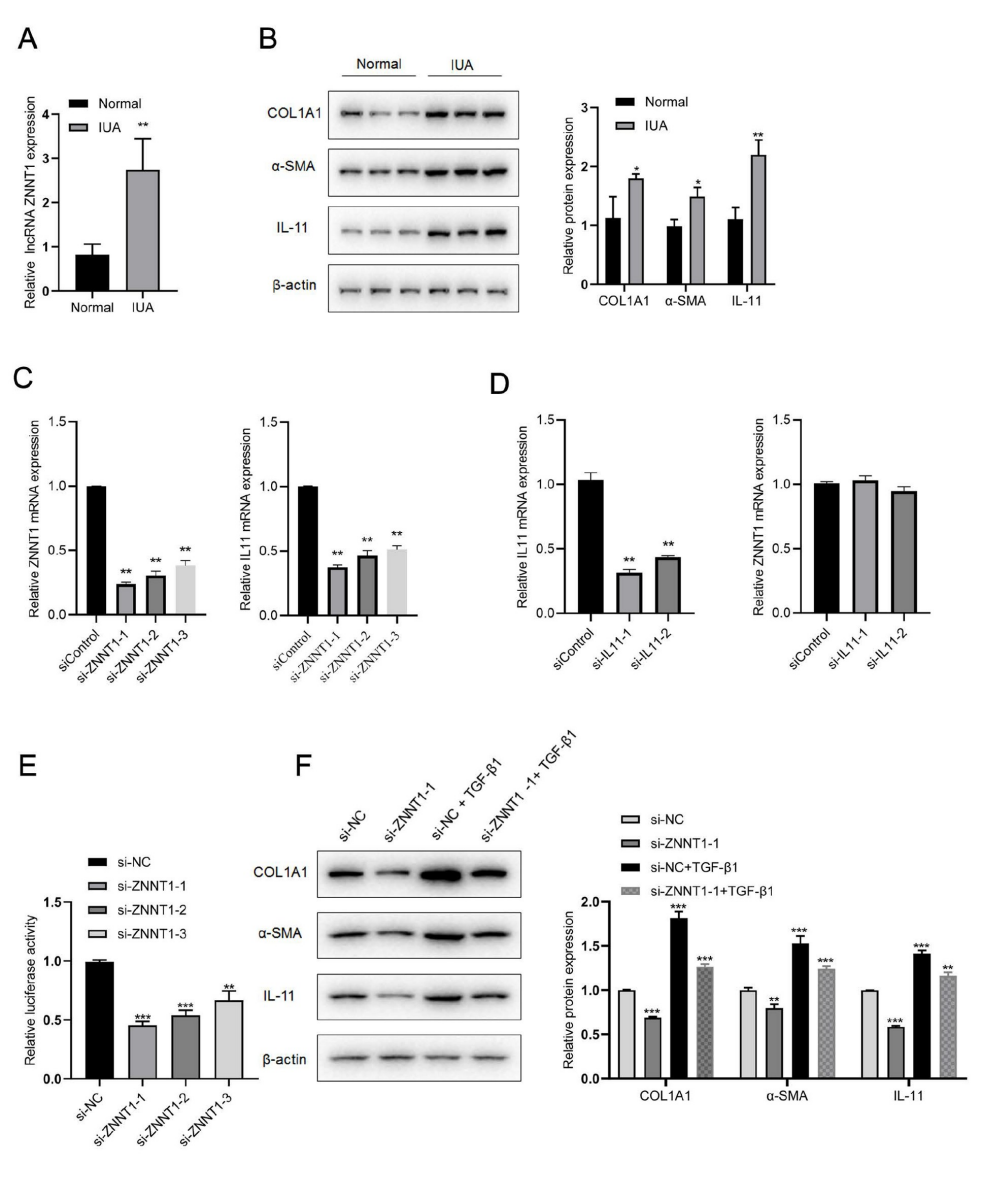
Figure 2 Silencing of lncRNA *ZNNT1* regulates IL11 expression to suppress endometrial fibrosis (A) LncRNA ZNNT1 expression was significantly elevated in IUA patient samples (n=10) compared to control samples. Data are presented as the mean±SEM. **P<0.01 vs normal controls. (B) COL1A1, α-SMA, and IL11 expression levels were significantly higher in IUA samples than in control samples, as measured by western blot analysis. *P<0.05, **P<0.01 vs normal controls. (C) Relative ZNNT1 mRNA expression (left) and relative IL11 mRNA expression (right) after si-ZNNT1 transfection into primary ESCs. **P<0.01 vs siControl. (D) Relative IL11 mRNA expression (left) and relative ZNNT1 mRNA expression (right) after si-IL11 transfection into primary ESCs. **P<0.01 vs siControl. (E) IL11 luciferase activity was assessed by dual-luciferase reporter assay, which indicated that ZNNT1 silencing suppressed IL11 expression. **P<0.01, ***P<0.001 vs si-NC. (F) Downregulation of COL1A1, α SMA, and IL11 protein expressions in si-ZNNT1-transfected ESCs treated with TGF-β1. **P<0.01, ***P<0.001 vs the si-NC or si-NC+TGF-β1 group. Data are presented as the mean±SEM. IUA, intrauterine adhesion.

In summary, the present study revealed that TGF-β1 treatment induced marked changes in the expressions of both lncRNAs and mRNAs in ESCs in endometrial fibrosis. Specific lncRNAs, such as ZNNT1, LOC101929577, LOC105370360, and LUCAT1, may regulate EMT induction in ESCs through their respective target mRNAs, including IL11, IGF2, COL1A2, and AREG, thus modulating the TGF-β signaling pathway. Of note, the lncRNA ZNNT1-IL-11 axis was found to be an important regulator of EMT induction and endometrial fibrosis, providing a foundation for future experimental research. Overall, the dysregulated lncRNAs and mRNAs identified herein may represent viable targets for preventing and treating IUA.

## Supporting information

23614Supplementary_tables_and_figures

## Supplementary Data

Supplementary data is available at *Acta Biochimica et Biophysica Sinica* online.
