# Supplementary material for: Integrated analysis of endometrial stromal cell long noncoding RNA and mRNA expression profiles associated with TGF-β1-induced fibrosis: lncRNA and mRNA expression profiles associated with TGF-β1-induced fibrosis in ESCs
Source: Acta Biochim Biophys Sin (Shanghai). 2024 Apr 18;56(6):952–5. doi: 10.3724/abbs.2024052 (PMC11214950; doi:10.3724/abbs.2024052)
Supplement: 23614Supplementary_tables_and_figures [file 23614Supplementary_tables_and_figures.pdf]

**Supplementary Table S1. The top 20 upregulated and downregulated DE-mRNAs**

| Upregulated gene | $Log_2FC$ | Q-value | Downregulated gene | $Log_2FC$ | Q-value |
|---|---|---|---|---|---|
| LOC100128242 | 6.80 | $2.77\times10^{-13}$ | COMMD3-BMI1 | -5.91 | $1.93\times10^{-7}$ |
| WRB-SH3BGR | 6.47 | $8.42\times10^{-11}$ | ZNF660-ZNF197 | -5.12 | $1.58\times10^{-4}$ |
| LOC102723728 | 6.31 | $8.51\times10^{-10}$ | BGIG9606_55019 | -4.38 | $7.54\times10^{-3}$ |
| BGIG9606_54690 | 5.92 | $1.15\times10^{-7}$ | INS-IGF2 | -4.28 | $1.12\times10^{-2}$ |
| ZNF816-ZNF321P | 4.15 | $2.87\times10^{-4}$ | BGIG9606_54773 | -4.17 | $1.70\times10^{-2}$ |
| CNTF | 4.03 | $2.47\times10^{-2}$ | TRIM72 | -4.03 | $2.67\times10^{-2}$ |
| ECT2L | 3.97 | $7.62\times10^{-4}$ | ADORA2A | -3.99 | $3.07\times10^{-2}$ |
| KLRC4-KLRK1 | 3.96 | $3.11\times10^{-2}$ | CHRNA7 | -3.68 | $4.39\times10^{-3}$ |
| LIMS3 | 3.95 | $3.17\times10^{-2}$ | KCNRG | -3.49 | $4.41\times10^{-4}$ |
| LAPTM5 | 3.43 | $1.27\times10^{-2}$ | IHH | -3.36 | $1.88\times10^{-2}$ |
| LOC101928635 | 3.29 | $2.21\times10^{-2}$ | NUTM1 | -3.36 | $1.88\times10^{-2}$ |
| GOLGA6A | 3.24 | $3.34\times10^{-2}$ | LOC112268305 | -3.20 | $3.26\times10^{-2}$ |
| LAD1 | 3.14 | $3.77\times10^{-2}$ | EMID1 | -3.20 | $3.26\times10^{-2}$ |
| ARL10 | 2.78 | $1.12\times10^{-2}$ | RIMBP3C | -2.98 | $3.75\times10^{-3}$ |
| PRR35 | 2.77 | $1.03\times10^{-2}$ | SDCBP2 | -2.89 | $1.48\times10^{-3}$ |
| ZNF117 | 2.77 | $3.61\times10^{-2}$ | EFHC2 | -2.84 | $8.19\times10^{-2}$ |
| BGIG9606_44121 | 2.67 | $1.74\times10^{-2}$ | BGIG9606_54910 | -2.77 | $2.05\times10^{-2}$ |
| TSPAN18 | 2.67 | $2.40\times10^{-4}$ | BGIG9606_39706 | -2.62 | $4.59\times10^{-9}$ |
| DSC2 | 2.55 | $2.87\times10^{-2}$ | STAB1 | -2.62 | $2.37\times10^{-2}$ |
| MMP7 | 2.55 | $2.87\times10^{-2}$ | BGIG9606_55237 | -2.49 | $1.41\times10^{-2}$ |

**Supplementary Table S2. The top 20 upregulated and downregulated DE-lncRNAs**

| Upregulated lncRNA | $Log_2$ FC | Q-value | Downregulated lncRNA | $Log_2$ FC | Q-value |
|---|---|---|---|---|---|
| BGIG9606_53904 | 11.41 | $1.26\times10^{-7}$ | BGIG9606_53941 | -5.10 | $2.11\times10^{-3}$ |
| PWAR6 | 6.23 | $2.52\times10^{-9}$ | LOC103344931 | -4.76 | $1.29\times10^{-3}$ |
| C8orf31 | 6.12 | $1.06\times10^{-8}$ | BGIG9606_55245 | -4.65 | $2.25\times10^{-3}$ |
| BGIG9606_47709 | 5.77 | $6.29\times10^{-3}$ | TNRC17 | -4.37 | $7.94\times10^{-3}$ |
| BGIG9606_67818 | 5.68 | $1.33\times10^{-6}$ | BGIG9606_55067 | -4.36 | $8.41\times10^{-3}$ |
| LOC100294145 | 5.35 | $7.55\times10^{-9}$ | BGIG9606_49204 | -4.16 | $1.74\times10^{-2}$ |
| EEF1E1-BLOC1S5 | 5.33 | $6.11\times10^{-10}$ | LOC101928673 | -4.15 | $1.85\times10^{-2}$ |
| BGIG9606_55961 | 5.21 | $6.66\times10^{-5}$ | LINC01299 | -4.02 | $2.77\times10^{-2}$ |
| LOC102724875 | 4.97 | $3.14\times10^{-4}$ | BGIG9606_50502 | -3.87 | $3.59\times10^{-7}$ |
| BGIG9606_54025 | 4.75 | $9.32\times10^{-3}$ | BGIG9606_45684 | -3.84 | $4.69\times10^{-2}$ |
| BGIG9606_49002 | 4.64 | $2.08\times10^{-3}$ | BGIG9606_52788 | -3.84 | $4.69\times10^{-2}$ |
| BGIG9606_52511 | 4.63 | $2.21\times10^{-3}$ | BGIG9606_55110 | -3.84 | $4.69\times10^{-2}$ |
| BGIG9606_65918 | 4.55 | $3.18\times10^{-3}$ | BGIG9606_56455 | -3.84 | $4.69\times10^{-2}$ |
| BGIG9606_55277 | 4.43 | $5.60\times10^{-3}$ | BGIG9606_62127 | -3.84 | $4.69\times10^{-2}$ |
| BGIG9606_54140 | 4.34 | $8.03\times10^{-3}$ | BGIG9606_65439 | -3.84 | $4.69\times10^{-2}$ |
| BGIG9606_55819 | 4.18 | $1.47\times10^{-2}$ | LOC105370670 | -3.84 | $4.69\times10^{-2}$ |
| BGIG9606_54213 | 4.18 | $1.49\times10^{-2}$ | BGIG9606_37552 | -3.66 | $4.62\times10^{-2}$ |
| BGIG9606_56300 | 4.14 | $1.74\times10^{-2}$ | LOC107985401 | -3.65 | $3.00\times10^{-3}$ |
| BGIG9606_50205 | 4.14 | $1.74\times10^{-2}$ | LOC105377018 | -3.62 | $6.00\times10^{-3}$ |
| BGIG9606_55060 | 4.04 | $2.36\times10^{-2}$ | BGIG9606_53894 | -3.58 | $5.31\times10^{-4}$ |

**Supplementary Table S3. Sequences of primers used for qPCR validation**

| Name | Primer sequence |
|---|---|
| *ZNNT1* | F: 5'-ACCAGTCTGCCACAAAGAGA-3' |
| | R: 5'-TTTCAGAAGCGGAGGACACT-3' |
| *LOC100129503* | F: 5'-TCCTGGAGAGAACAGTGCAG-3' |
| | R: 5'-TATCAGTGCTGCTCCCAACA-3' |
| *LOC101927267* | F: 5'-TCCAGGCTGGTATCGAACTC-3' |
| | R: 5'-TGCTTCGCCTCTAGGATCTC-3' |
| *LOC101929577* | F: 5'-CAGCCTAGCAGATGCCAGAT-3' |
| | R: 5'-CTAGTGGCCACATCAGCAAC-3' |
| *LOC105370360* | F: 5'-GGTTTCGAGCTATGCTTGGG-3' |
| | R: 5'-TCTCTCATGCCACAGTCAGG-3' |
| *LUCAT1* | F: 5'-GTGCCTGTACAGTTGTGTCC-3' |
| | R:5'-TTATCCTCGGGTTGCCTCTG-3' |
| *BGIG9606_58128* | F: 5'-AGATAGTTGGGTGGTTGGTGT-3' |
| | R: 5'-CACAACACTAAGGGACGAACC-3' |
| *IL11* | F: 5'-CTGAGCCTGTGGCCAGATA-3' |
| | R: 5'-AGCTGGGAATTTGTCCCTCA-3' |
| *IL21R* | F: 5'-GAGTCCGAGGAGAAAGCTGA-3' |
| | R: 5'-GGGTCACTCCATTCACTCCA-3' |
| *IGF2* | F: 5'-GTGCTTCTCACCTTCTTGGC-3' |
| | R: 5'-CGGAAACAGCACTCCTCAAC-3' |

| | |
|---|---|
| *COL1A2* | F: 5'-GTGAAGATGGTCACCCTGGA-3' |
| | R: 5'-GCCTTTGAAGCCAGGAAGTC-3' |
| *SGK2* | F: 5'-GACCTGTACCACAAGAGGCT-3' |
| | R: 5'-GCCAATGGACTTGGACACAG-3' |
| *LAMA1* | F: 5'-TTCGCTTGCAACGCATTAGA-3' |
| | R: 5'-CATCCCATGGGCAGCTACTA-3' |
| *AREG* | F: 5'-GGTGCTGTCGCTCTTGATAC-3' |
| | R: 5'-TTCACGCTTCCCAGAGTAGG-3' |
| *GAPDH* | F: 5′ -GAAGGTGAAGGTCGGAGTC-3′ |
| | R: 5′ -GAAGATGGTGATGGGATTTC-3′ |

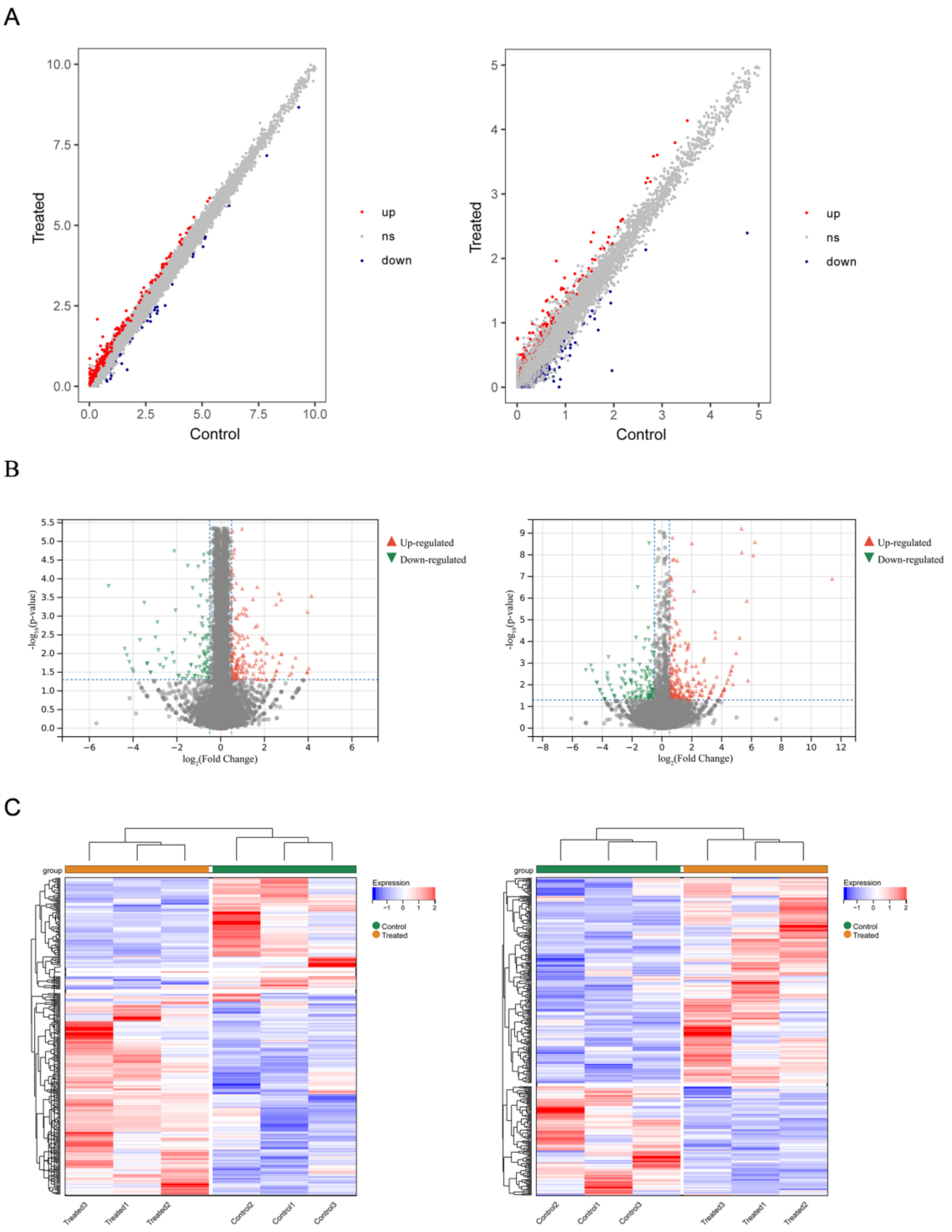


**Supplementary Figure S1. Identification of differential lncRNA and mRNA expression in TGF-β1-treated ESCs relative to control ESCs** Scatterplots comparing the expressions of mRNAs (left) and lncRNAs (right) between TGF-β1-treated and untreated ESCs. Normalized expression values are shown. (B) Volcano plots comparing the expressions of mRNAs (left) and lncRNAs (right)

between TGF-β1-treated and untreated ESCs (C) Heatmaps showing significant DE-mRNAs (left) and DE-lncRNAs (right). Red represents upregulation, blue represents downregulation, and gray represents no significant change.

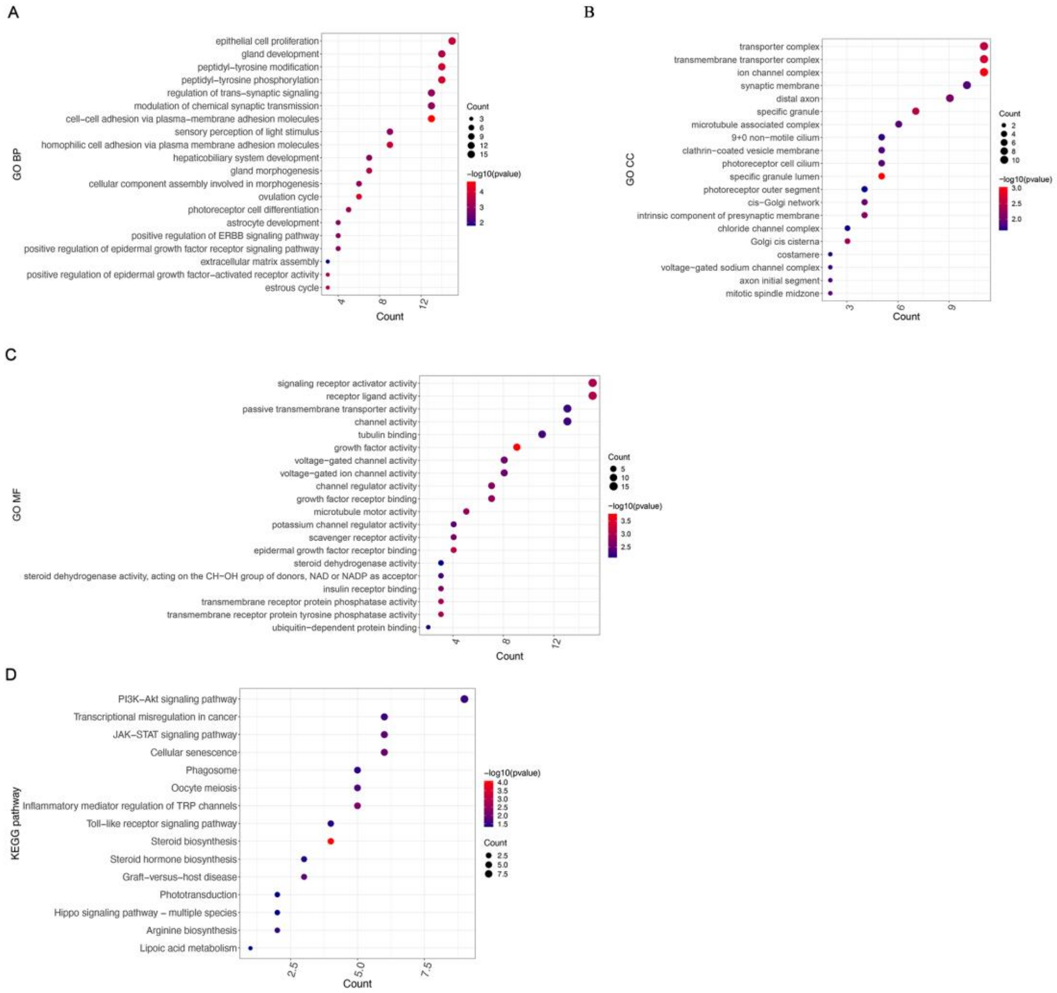


**Supplementary Figure S2. Functional enrichment of DE-mRNAs** (A-C) Top 20 GO terms in the biological process (A), cellular component (B), and molecular function (C) categories. (D) The top 15 most-enriched KEGG pathways associated with identified DE-mRNAs. The x-axis corresponds to enriched gene counts.

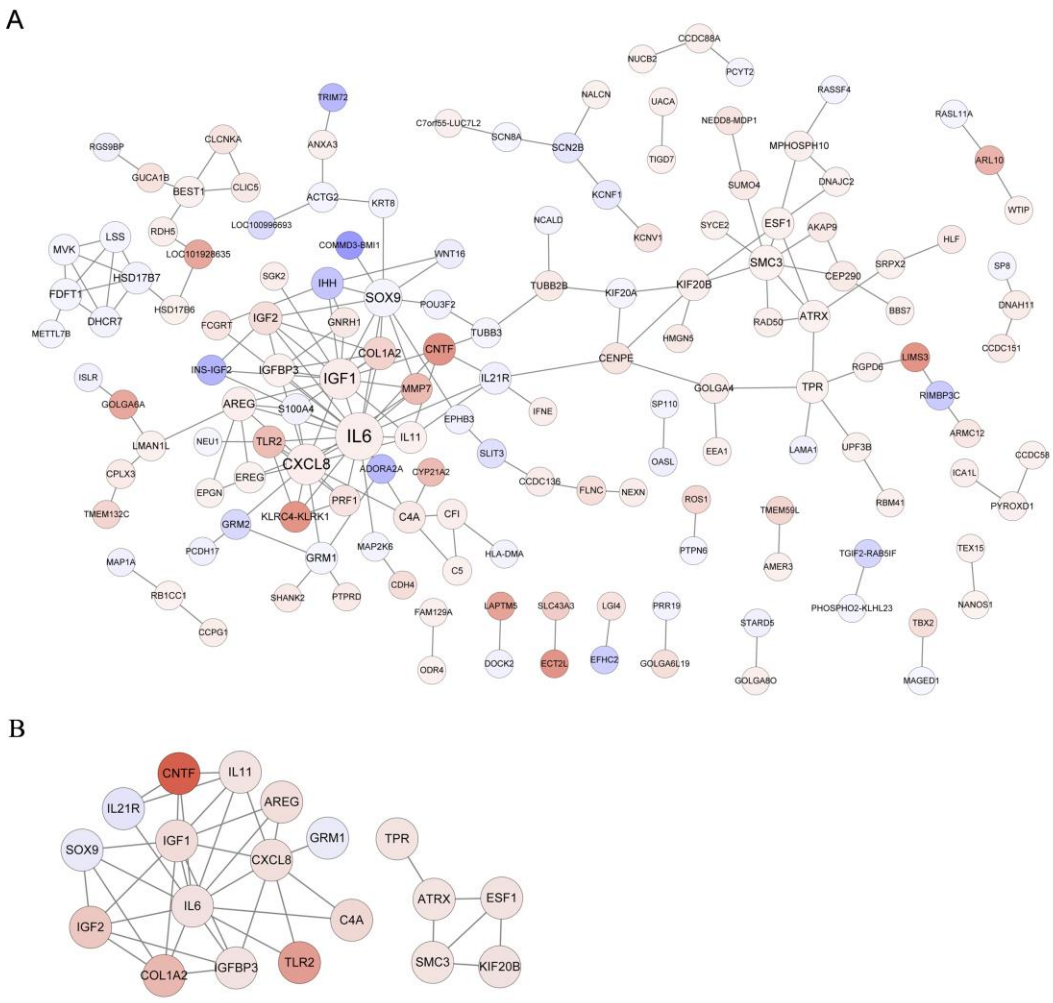


**Supplementary Figure S3. PPI network analysis of endometrial fibrosis-related DE-mRNAs** (A) PPI network based on the 327 DE-mRNAs between TGF-β1-treated and untreated ESCs. (B) Subnetwork of the top 20 DE-mRNAs showing the highest connectivity values in the PPI network. Red and blue nodes correspond to upregulated and downregulated mRNAs respectively, with edges serving to link interacting targets. Node size is proportional to the degree of connectivity and the intensity of node color is proportional to the corresponding gene expression fold-change value.

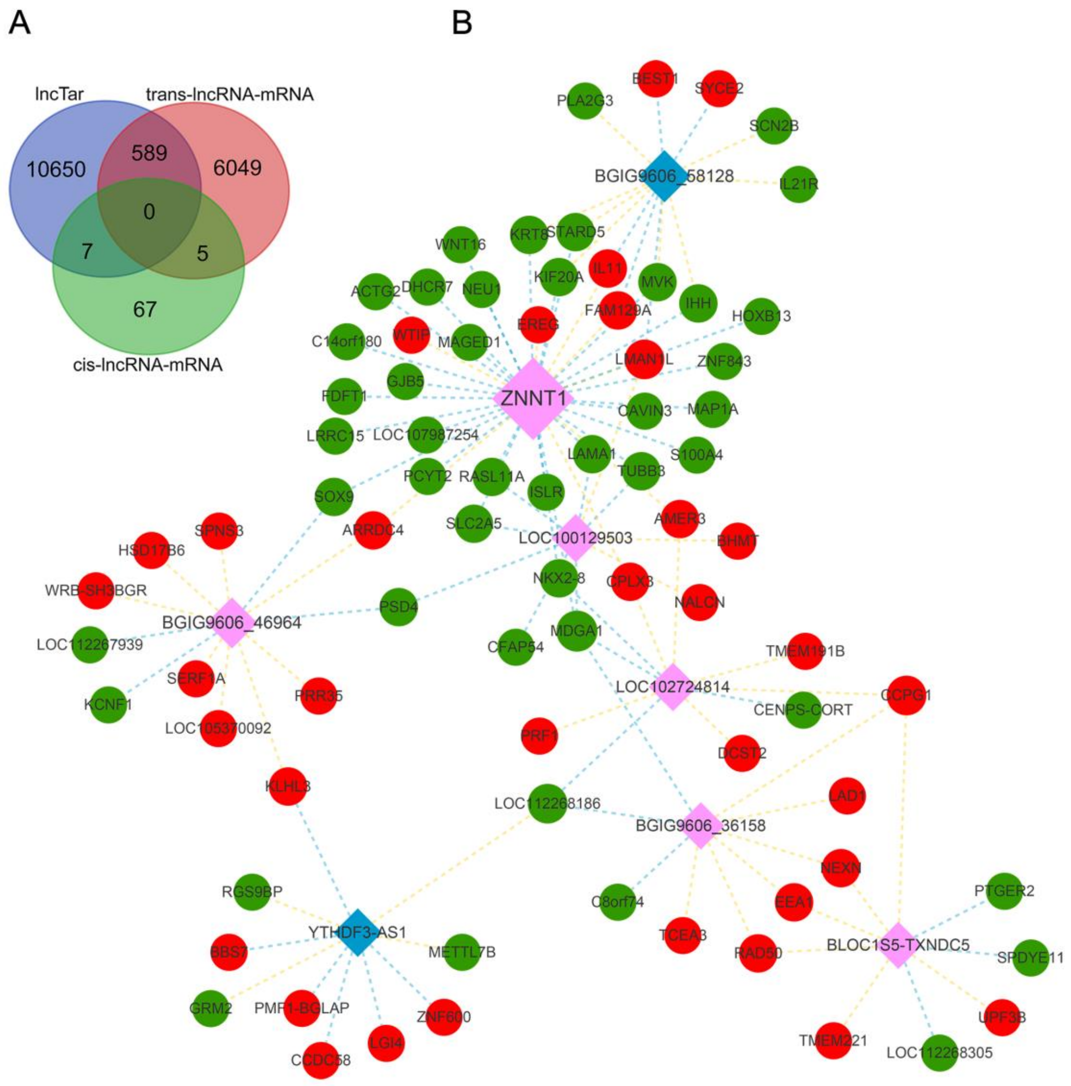


**Supplementary Figure S4. LncRNA-mRNA regulatory network analyses** (A) Venn diagram showing overlap between the lncRNA-mRNA interaction pairs in the three datasets. (B) A lncRNA-mRNA interaction network for the top 8 DE-lncRNAs associated with the greatest number of mRNA targets. Red and green circular nodes represent upregulated and downregulated mRNAs, respectively, in TGF-β1-treated ESCs. Pin and blue diamond nodes represent upregulated and downregulated lncRNAs in TGF-β1-treated ESCs. Positive and negative correlations are denoted by yellow and light-blue lines respectively. Diamond node sizes are proportional to the number of target mRNAs associated with that lncRNA. *P*<0.05 was the threshold of significance when identifying mRNA targets of lncRNAs.

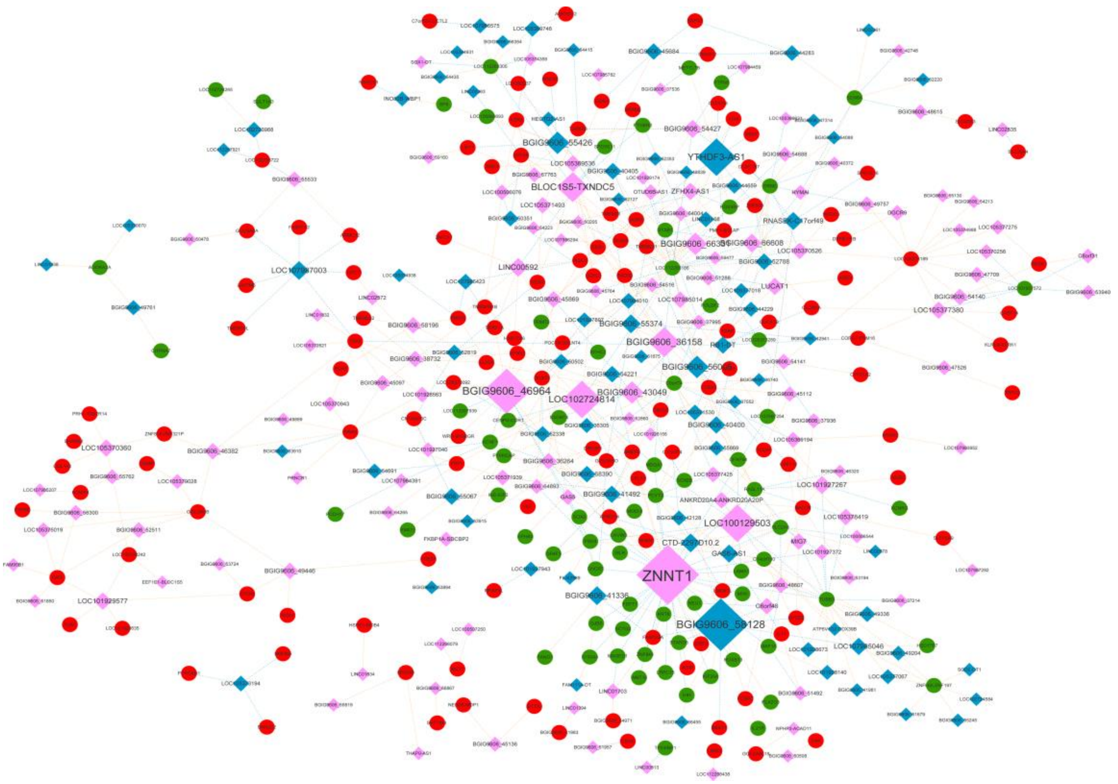


**Supplementary Figure S5. Development of a lncRNA-mRNA interaction network based on DE-lncRNAs and associated mRNA targets** Red and green circular nodes represent upregulated and downregulated mRNAs respectively in TGF-β1-treated ESCs. Pink and blue diamond nodes represent upregulated and downregulated lncRNAs respectively in TGF-β1-treated ESCs. Positive and negative correlations are denoted by yellow and light-blue lines respectively. Diamond node sizes are proportional to the number of target mRNAs associated with that lncRNA. $P<0.05$ was the threshold of significance when identifying mRNA targets of lncRNAs.
